# Supplementary material for: Optical phonons of SnSe(1−x)Sx layered semiconductor alloys
Source: Sci Rep. 2020 Jul 16;10:11761. doi: 10.1038/s41598-020-68744-2 (PMC7366649; doi:10.1038/s41598-020-68744-2)
Supplement: Supplementary file 1 — Supplementary information 1. [file 41598_2020_68744_MOESM1_ESM.pdf]

Supplementary Information for

Optical Phonons of $SnSe_{(1-x)}S_x$ Layered Semiconductor Alloys

Tharith Sriv[1,2], Thi Minh Hai Nguyen[3], Yangjin Lee[4,5], Soo Yeon Lim[1], Van Quang Nguyen[3], Kwanpyo Kim[4,5], Sunglae Cho[3] and Hyeonsik Cheong[1,*]

[1]Department of Physics, Sogang University, Seoul 04107, Korea
[2]Department of Physics, Royal University of Phnom Penh, Phnom Penh, Cambodia
[3]Department of Physics and Energy Harvest Storage Research Center (EHSRC), University of Ulsan, Ulsan 44610, Korea
[4]Department of Physics, Yonsei University, Seoul 03722, Korea
[5]Center for Nanomedicine, Institute for Basic Science (IBS), Seoul 03722, Korea
[*]Coresponding author: hcheong@sogang.ac.kr

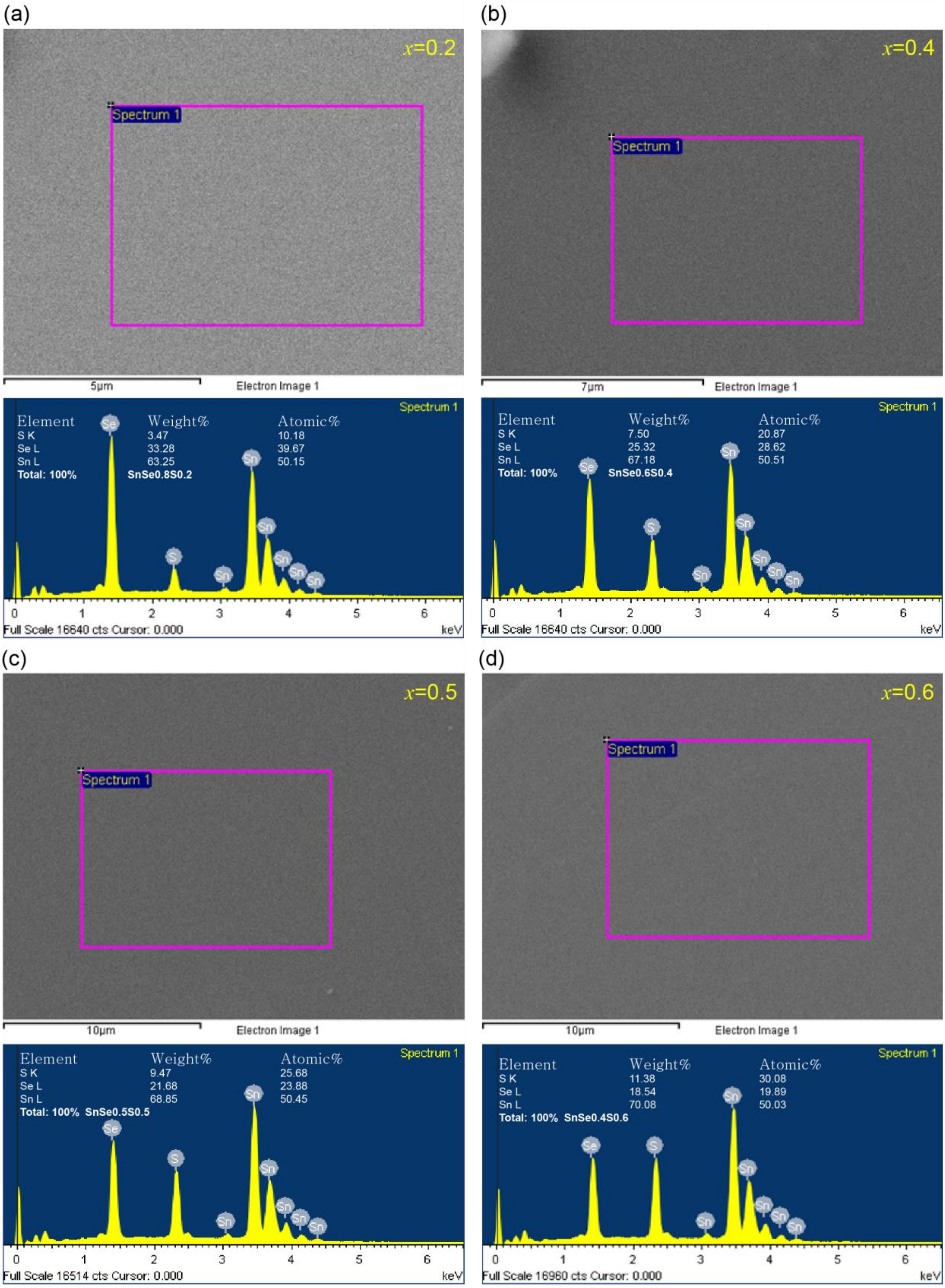

Figure S1. Energy dispersive X-ray spectroscopy (EDS) results of $SnSe_{(1-x)}S_x$ (x=0.2, 0.4, 0.5, 0.6) alloys along with SEM images showing where the EDS measurements were made.

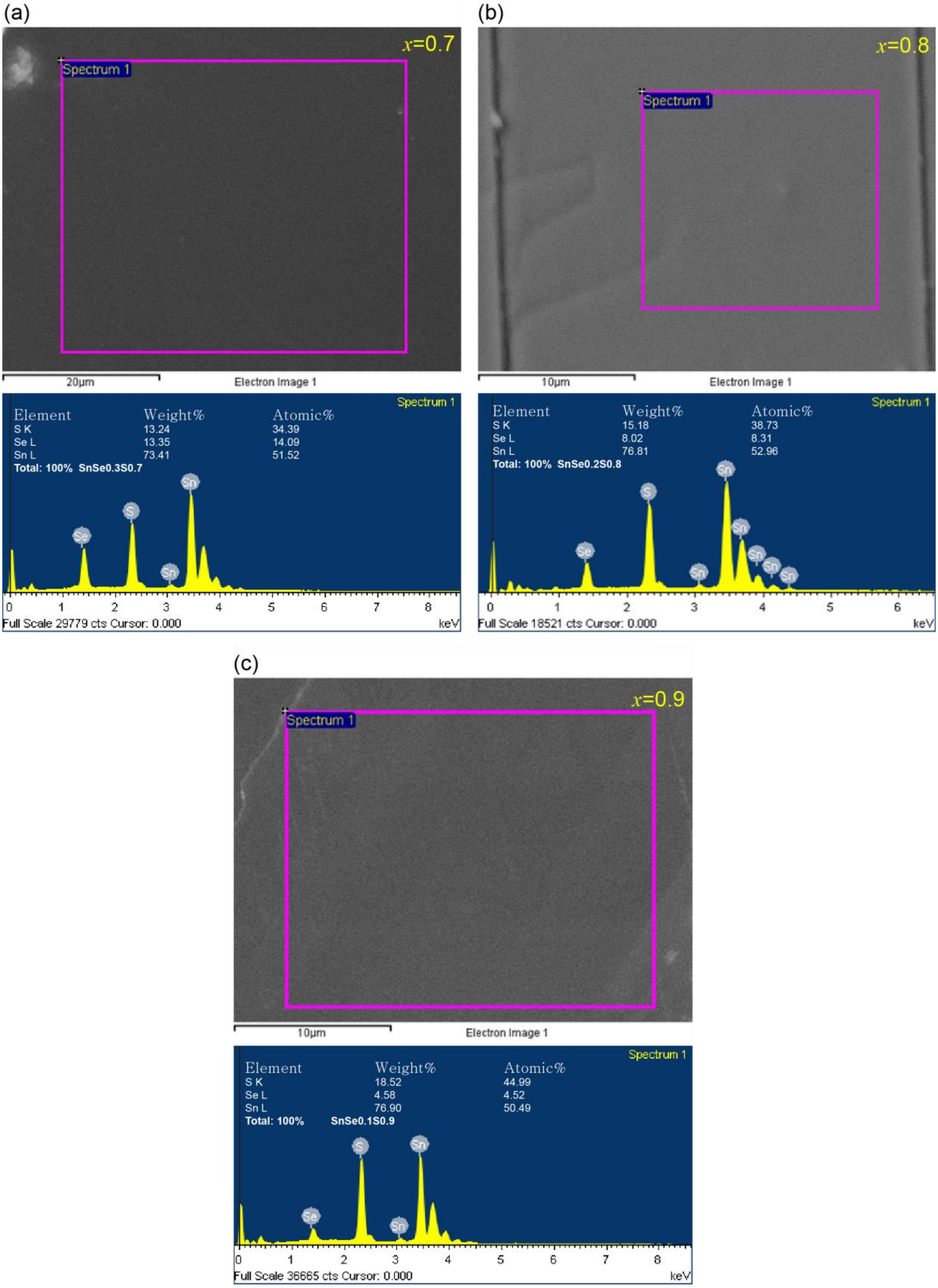

Figure S2. EDS results of $SnSe_{(1-x)}S_x$ (x=0.7, 0.8, 0.9) alloys along with SEM images showing where the EDS measurements were made.

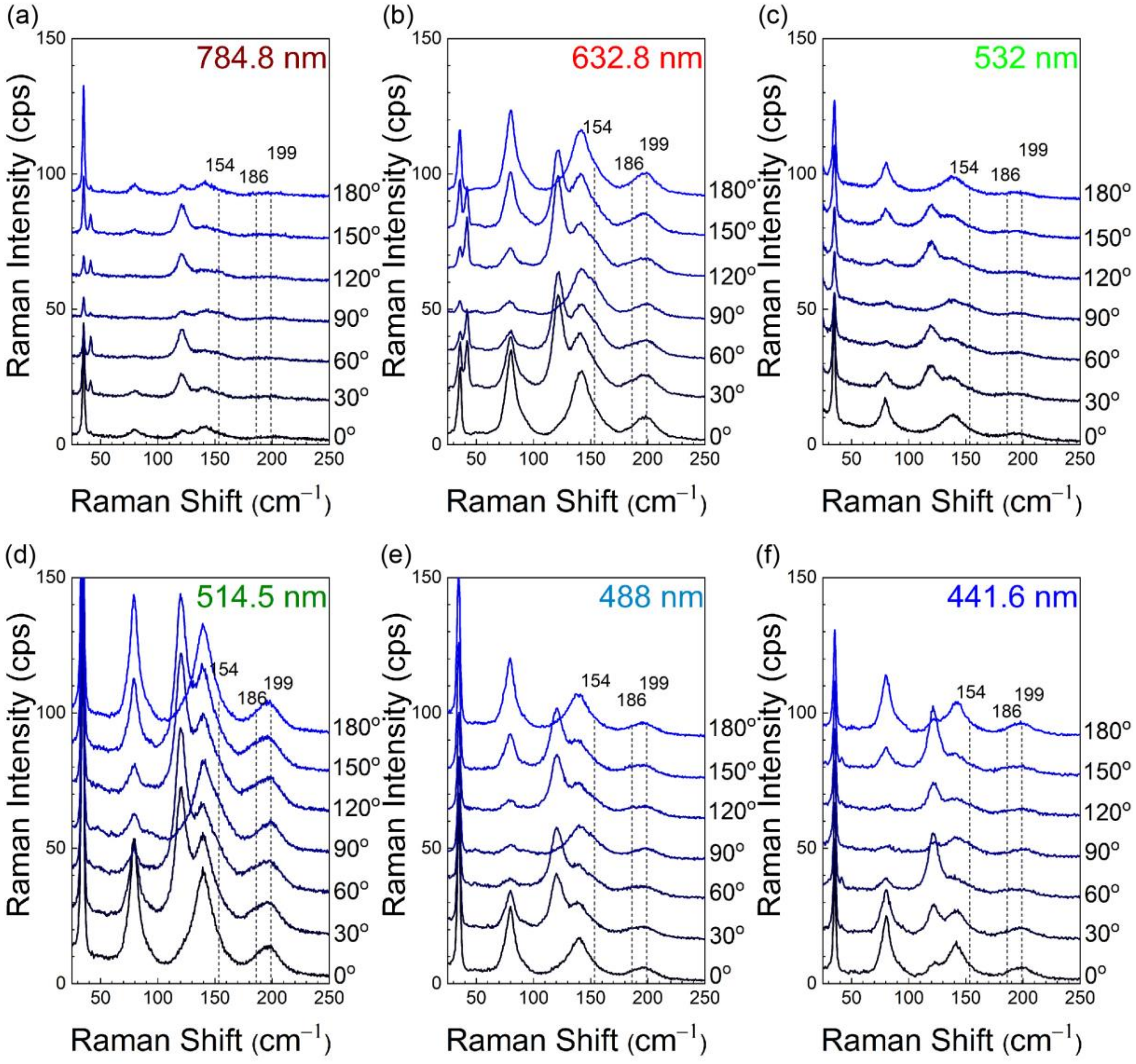

Figure S3. Polarization dependence of Raman spectra of $SnSe_{0.5}S_{0.5}$ alloys measured in parallel polarization configuration with six excitation wavelengths as indicated. The vertical gray dotted lines are guide for the eyes from comparison with fitting results (Figure 3) and show the position of weak peaks such as SnSe-like A_g^4 (~154 cm^{-1}), SnS-like B_{3g}^2 (~186 cm^{-1}) and SnS-like A_g^3 (~199 cm^{-1}), respectively.

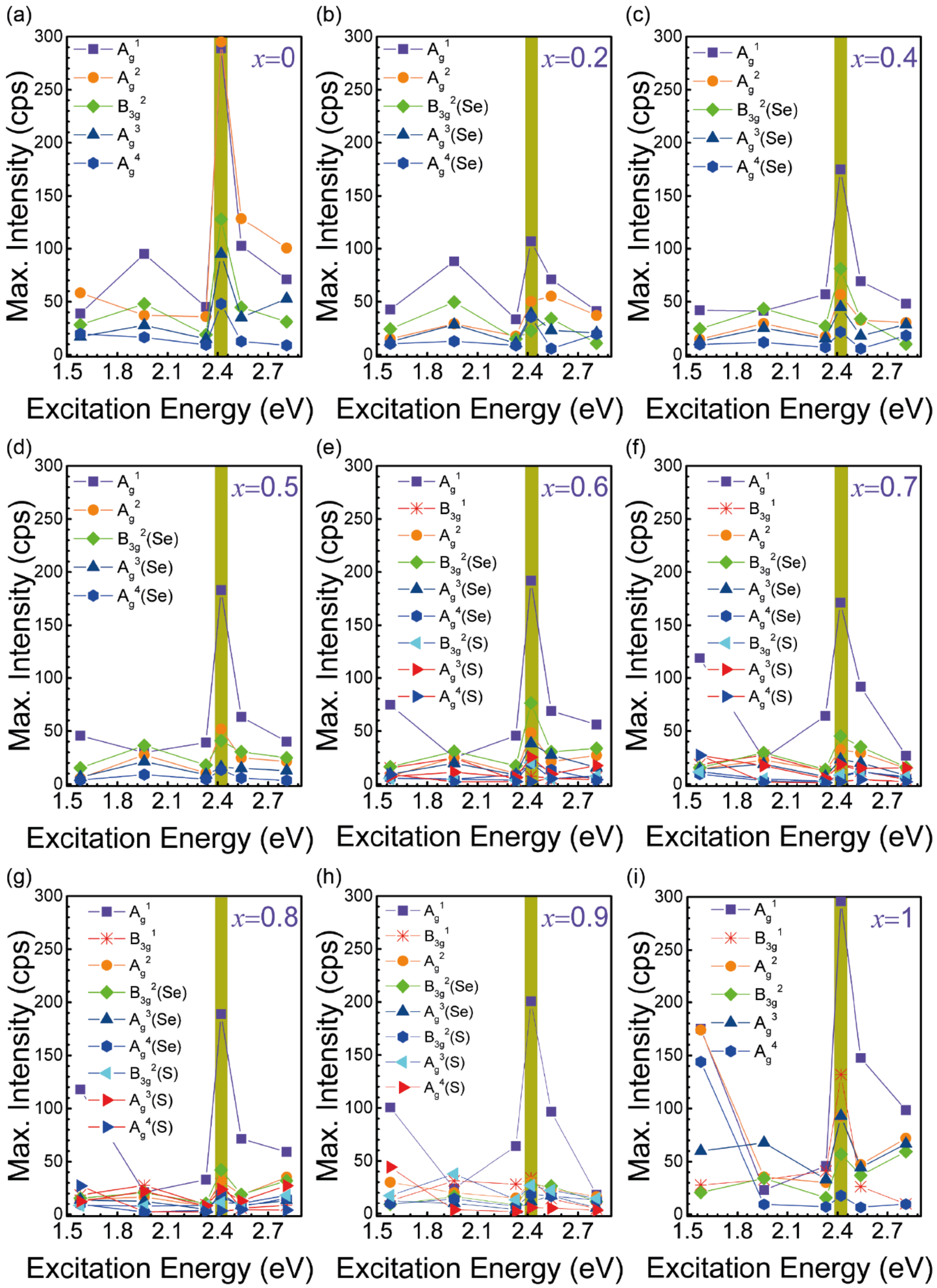

Figure S4. Excitation energy dependence of the maximum intensity of Raman peaks in the $SnSe_{(1-x)}S_x$ alloys. (Se) and (S) in parentheses refer to SnSe- and SnS-like modes, respectively.

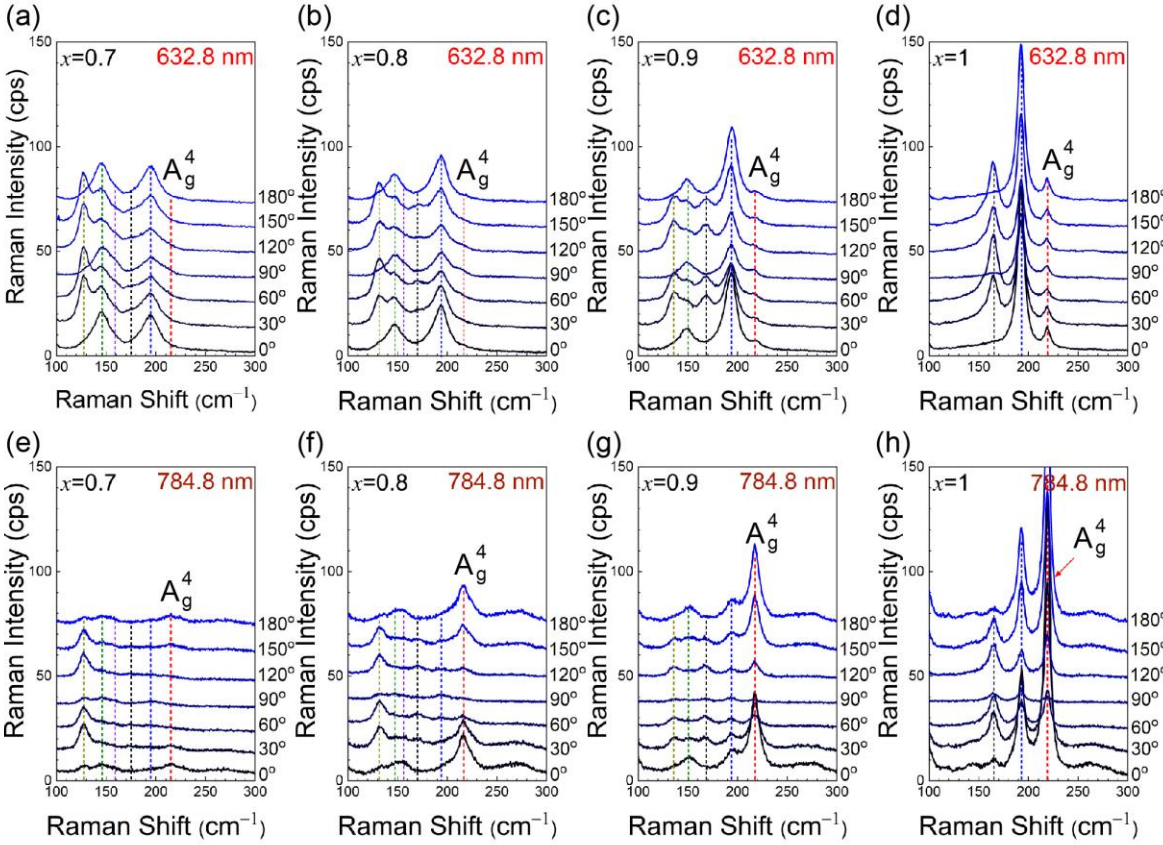

Figure S5. Comparison of polarization dependence of Raman spectra of $SnSe_{(1-x)}S_x$ (x=0.7, 0.8, 0.9, 1) single crystals measured in parallel polarization configuration with different excitation wavelengths (632.8- and 784.8-nm). The SnS-like A_g^4 peak (indicated by red dotted line) is strongly enhanced for the 784.8-nm (1.58 eV) excitation wavelength which is close to resonance with the band gap of SnS (~1.5 eV).

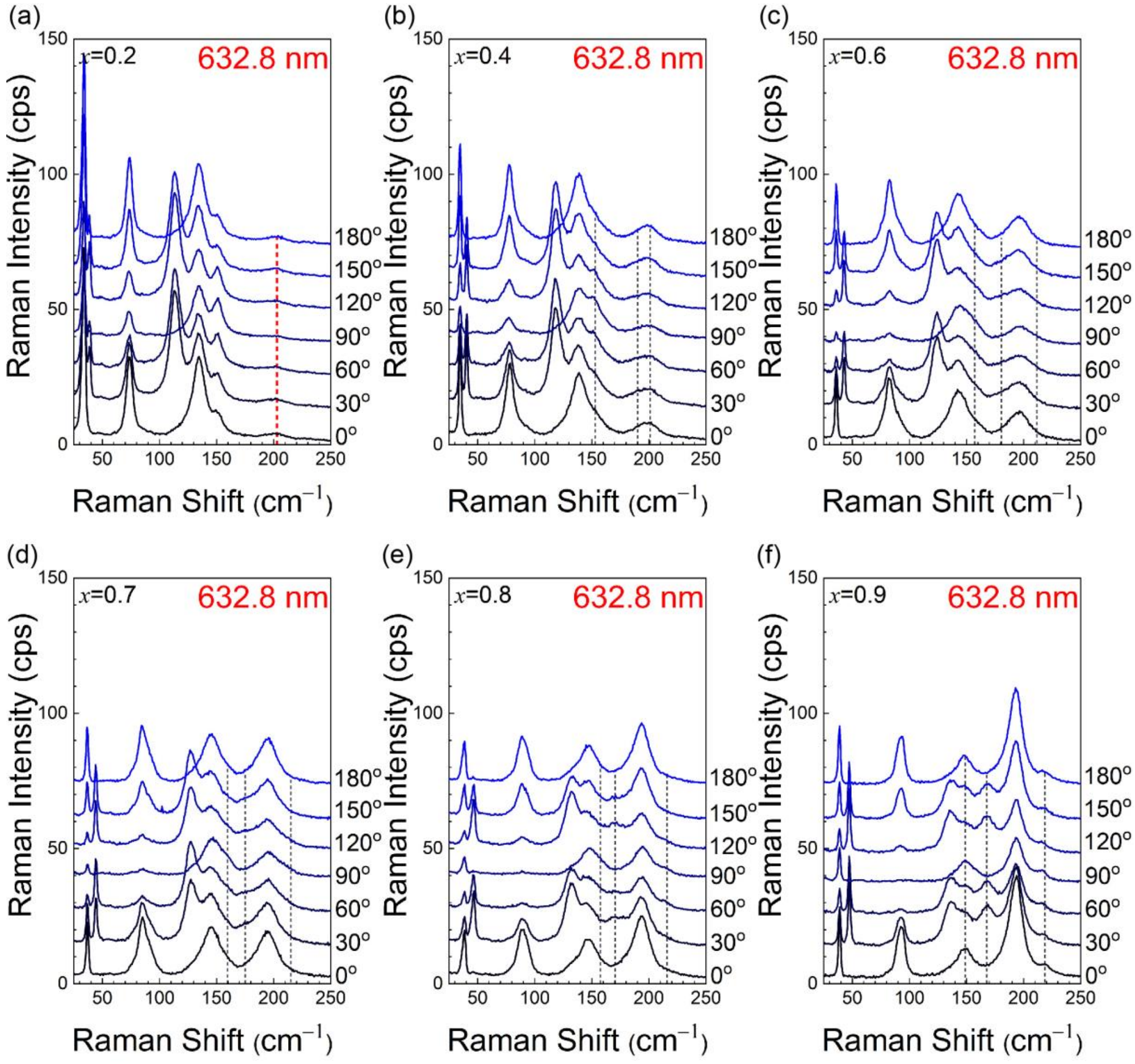

Figure S6. Polarization dependence of Raman spectra of $SnSe_{(1-x)}S_x$ (x=0.2, 0.4, 0.6, 0.7, 0.8, 0.9) single crystals measured in parallel polarization configuration by using 632.8 nm excitation wavelength. The red vertical dash line at ~203 cm^{-1} in $SnSe_{0.8}S_{0.2}$ alloy indicates the positions of overlapping Raman peaks, SnS-like B_{3g}^2 and A_g^3. Gray vertical dash lines at the position of weak modes in high frequency range are marked as guide for the eyes obtained from deconvolution of the spectra.

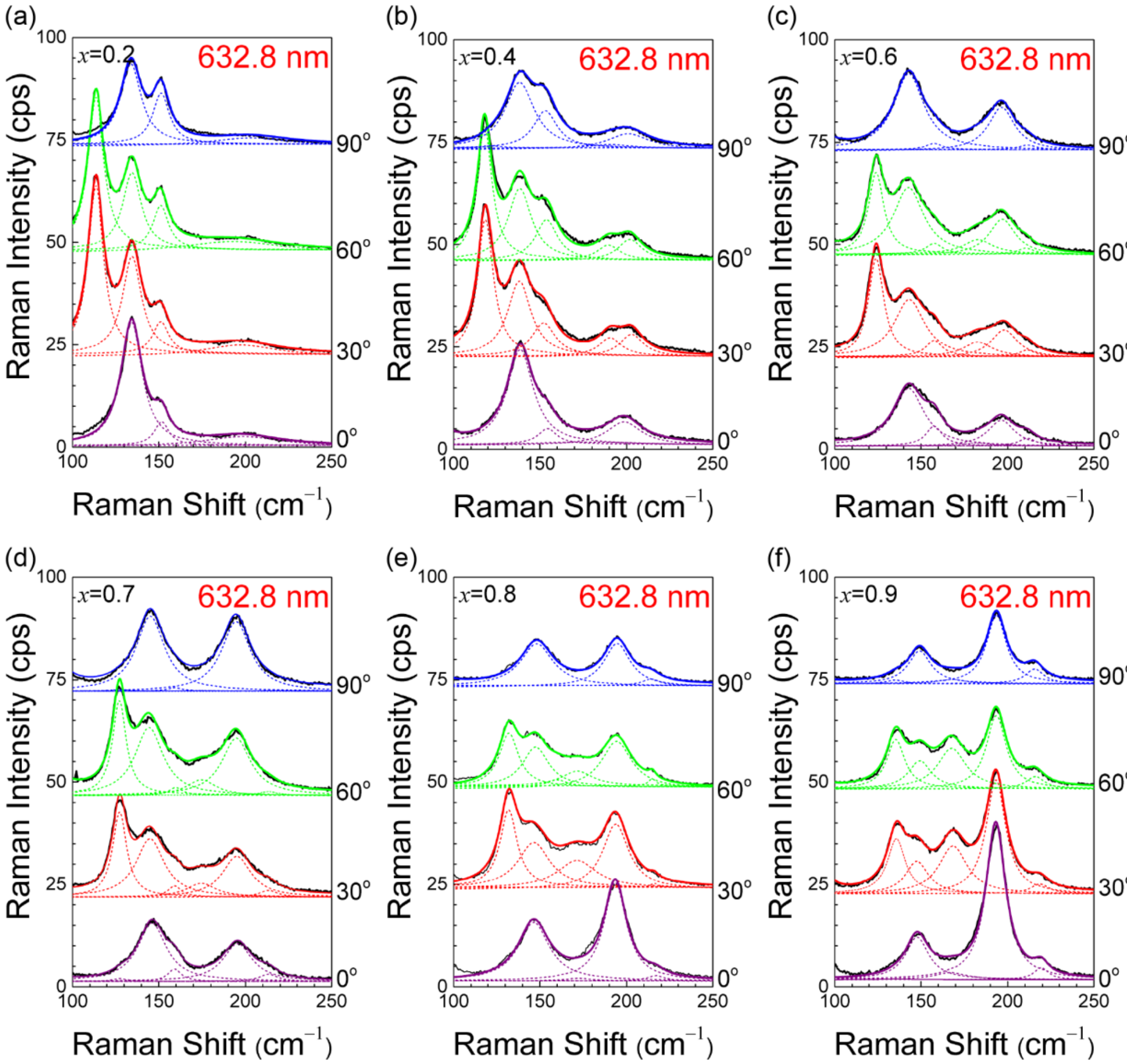

Figure S7. Deconvolution of Raman spectra of $SnSe_{(1-x)}S_x$ (x=0.2, 0.4, 0.6, 0.7, 0.8, 0.9) measured in parallel polarization configuration by using 632.8-nm excitation wavelength in several polarization directions as indicated. Deconvolution was done in OriginPro using Lorentzian functions to fit each peak in the Raman spectra. To further obtain polar plots in Figure S8, S9, S10 and elsewhere, integrated intensity (peak area) was used.

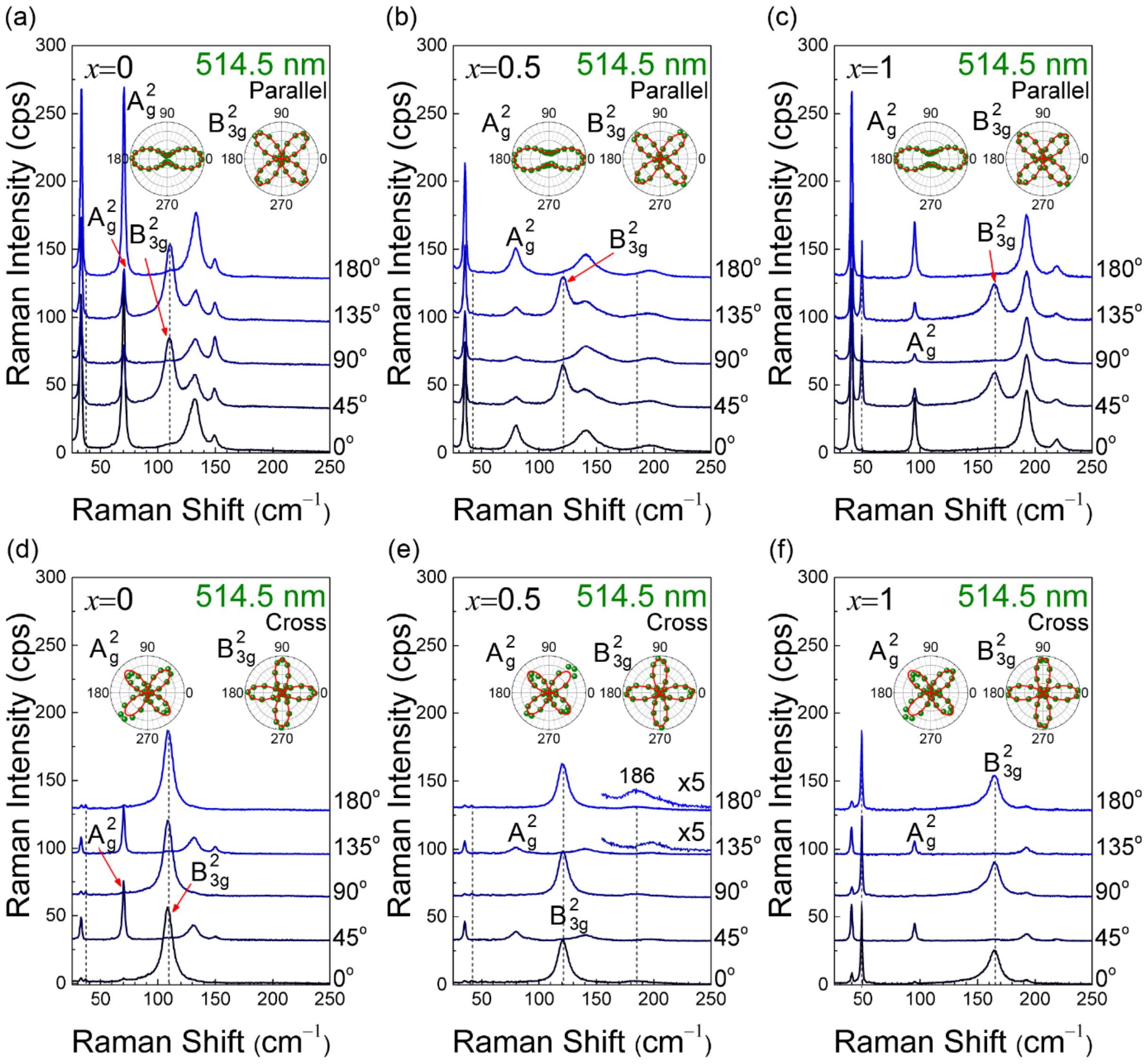

Figure S8. Comparison of polarization dependence of Raman spectra of SnSe, $SnSe_{0.5}S_{0.5}$ and SnS measured with 514.5-nm excitation wavelength in parallel and cross polarization configurations. Insets show the polarization dependence of the normalized Raman intensity of the A_g^2 and B_{3g}^2 modes, respectively. The curves represent best fits to the calculated polarization dependence of the Raman intensities using equation (1)-(4). The vertical dashed lines indicate the position of the B_{3g} modes in SnSe and SnS and the corresponding modes in the $SnSe_{0.5}S_{0.5}$ alloy.

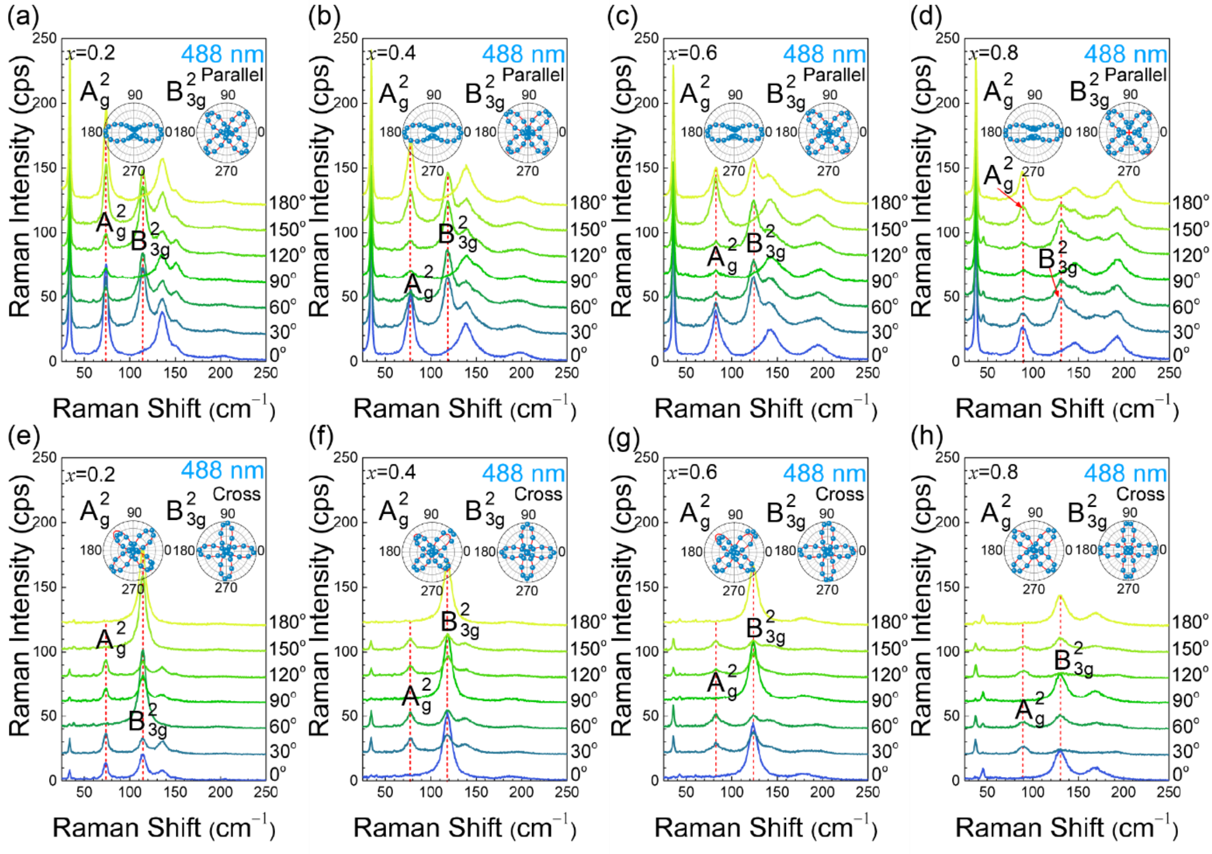

Figure S9. Comparison of polarization dependence of Raman spectra of $SnSe_{(1-x)}S_x$ (x=0.2, 0.4, 0.6, 0.8) measured in parallel and cross polarization configurations with 488-nm excitation wavelength. Insets are polar plots of the normalized Raman intensity of the A_g^2 and B_{3g}^2 modes. The curves represent best fits to the calculated polarization dependence of the Raman intensities using equation (1) and (2).

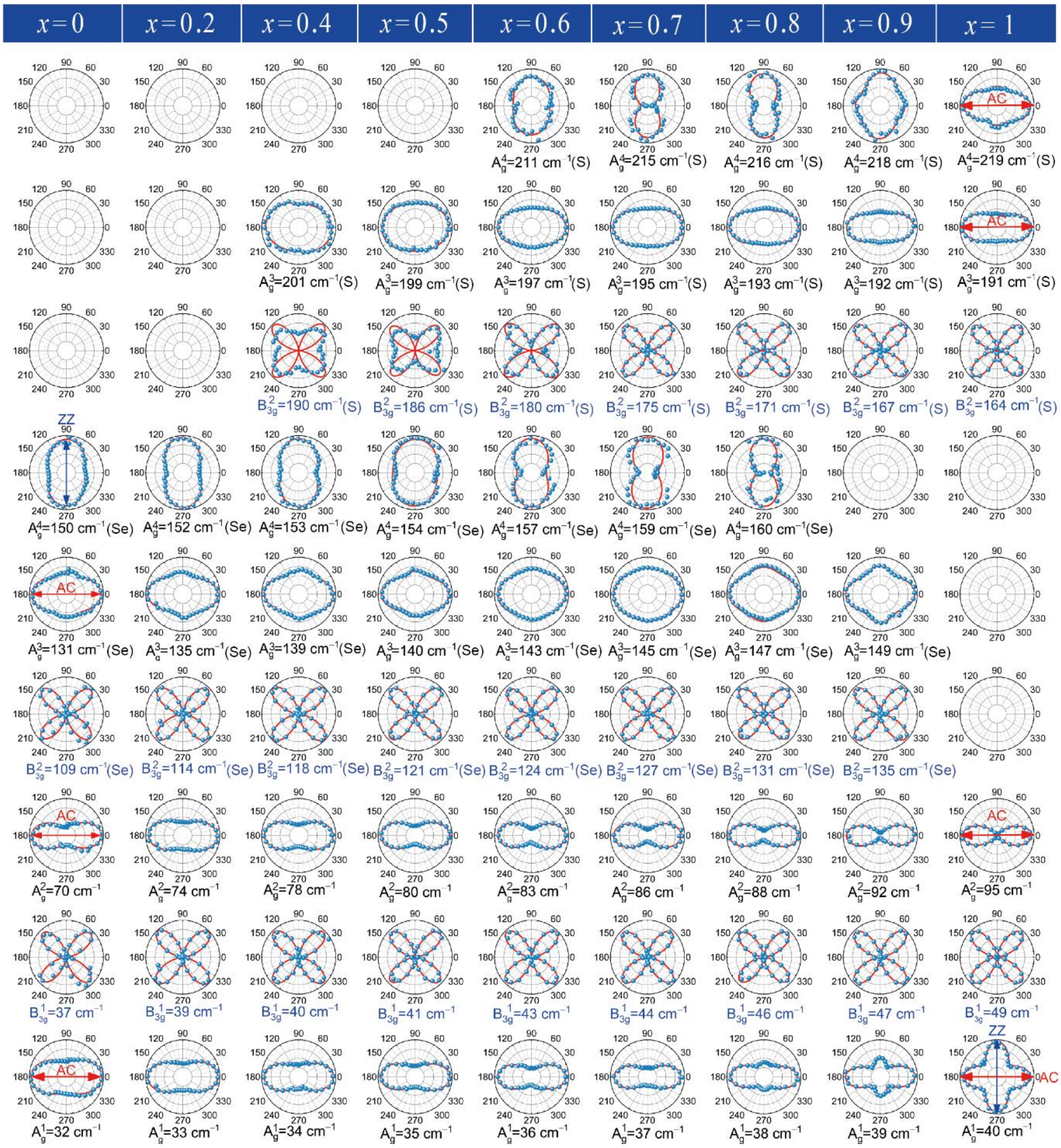

Figure S10. Polarization dependence of Raman modes of $SnSe_{(1-x)}S_x$ ($0 \le x \le 1$) single crystals measured by using 632.8-nm excitation wavelength in parallel polarization configuration. The curves represent best fits to the calculated polarization dependence of the Raman intensities of equation (1) and (2). The armchair (AC) direction is set to 0°. AC and zigzag (ZZ) directions are indicated by red and blue arrows, respectively. (Se) and (S) in parentheses refer to SnSe- and SnS-like modes, respectively.

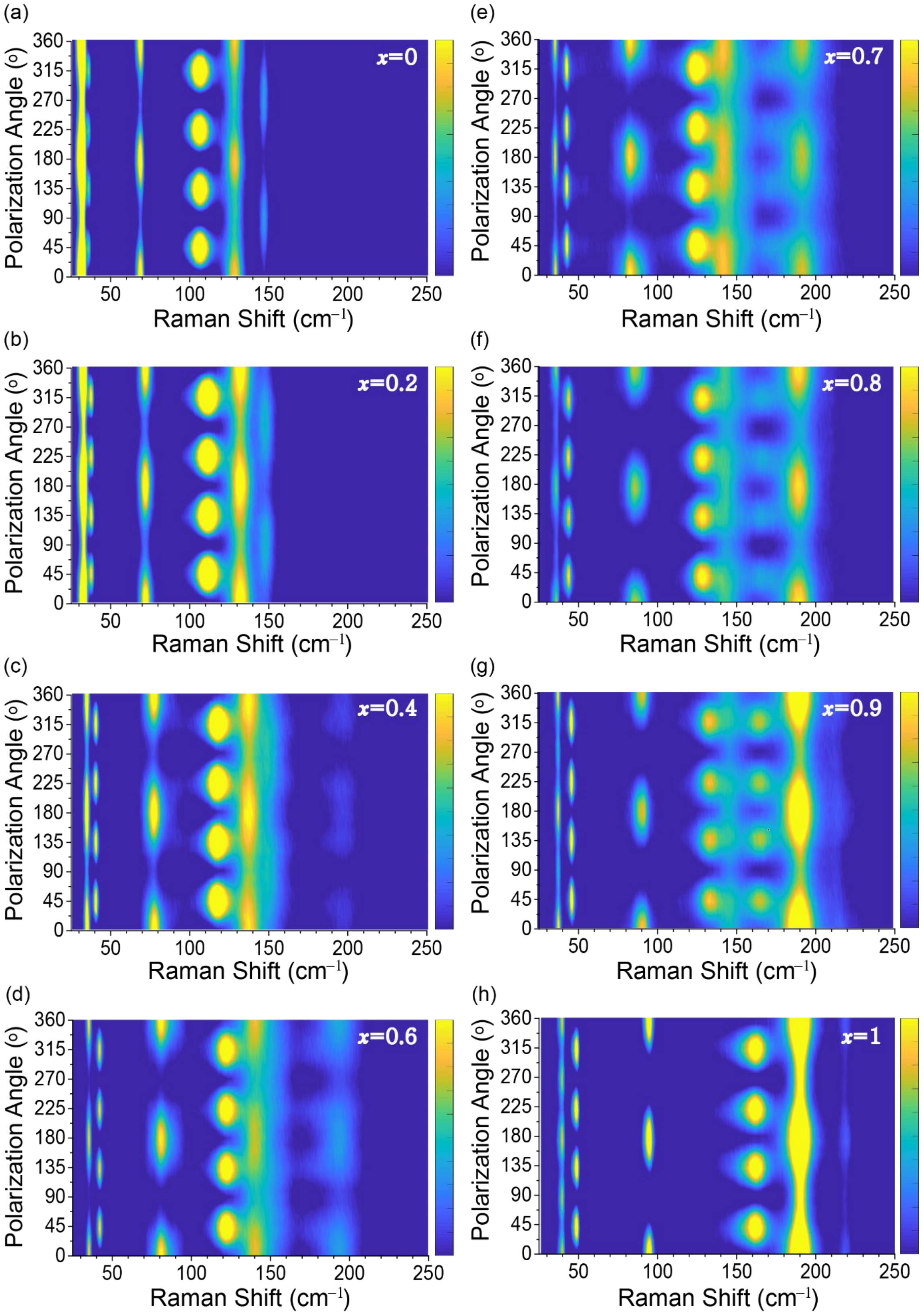

Figure S11. Polarization dependence of Raman spectrum intensity of $SnSe_{(1-x)}S_x$ ($0 \leq x \leq 1$) measured by using 632.8-nm excitation wavelength in parallel polarization configuration,

measured in 10° increments. The color bar indicates the normalized scale of the Raman intensity of each peak.

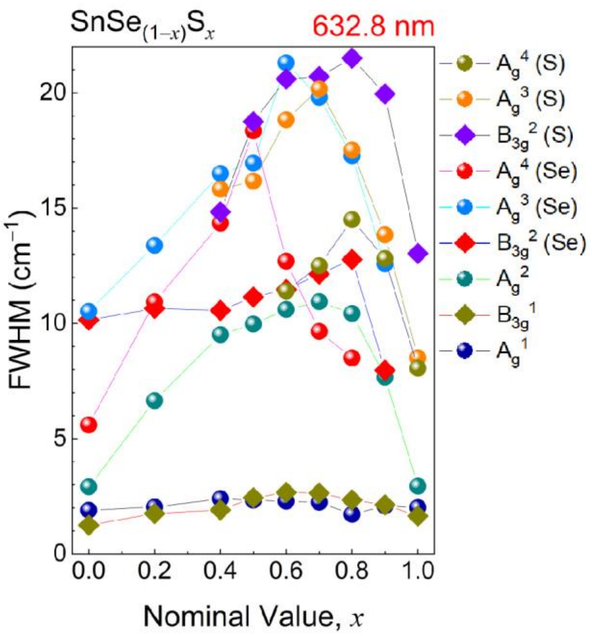

Figure S12. Full-width-at-half-maximum (FWHM) of each Raman peak in $SnSe_{(1-x)}S_x$ ($0 \leq x \leq 1$) as a function of the nominal value composition x measured with 632.8-nm excitation wavelength in parallel configuration. (Se) and (S) in parentheses refer to SnSe- and SnS-like modes, respectively.

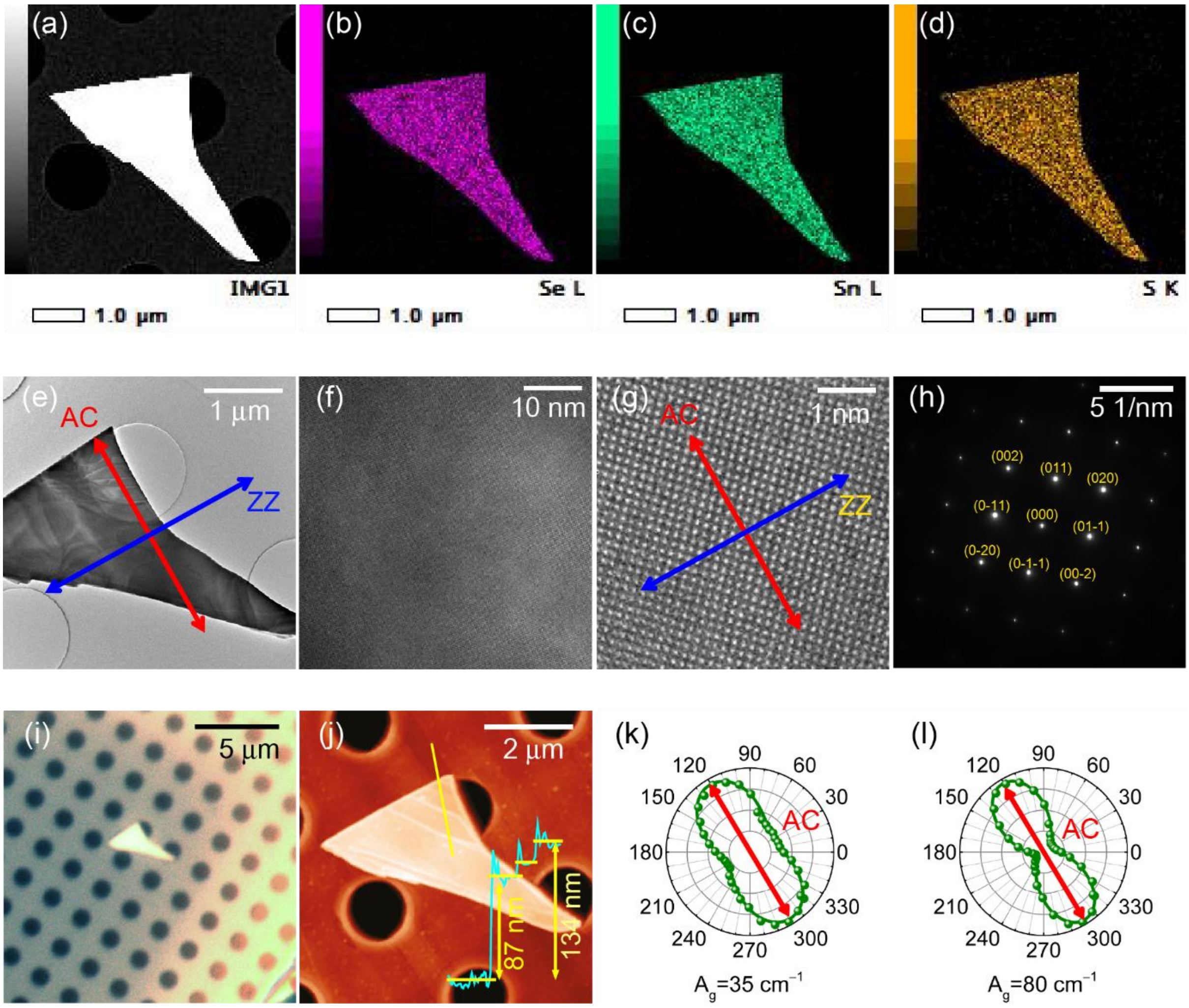

Figure S13. Comparison of transmission electron microscopy (TEM) and polarized Raman results to determine crystallographic directions of $SnSe_{0.5}S_{0.5}$. (a-d) EDS mapping results. (e, f) TEM image of the sample. (g) high-resolution TEM images with the zigzag and armchair orientations indicated. (h) SAED pattern. (i) optical and (j) AFM images of the sample, respectively. (k) and (l) polarization dependence of A_g^1 (35 cm^{-1}) and A_g^2 (80 cm^{-1}) modes in parallel polarization configuration showing that the armchair direction matches with the maximum intensity direction.

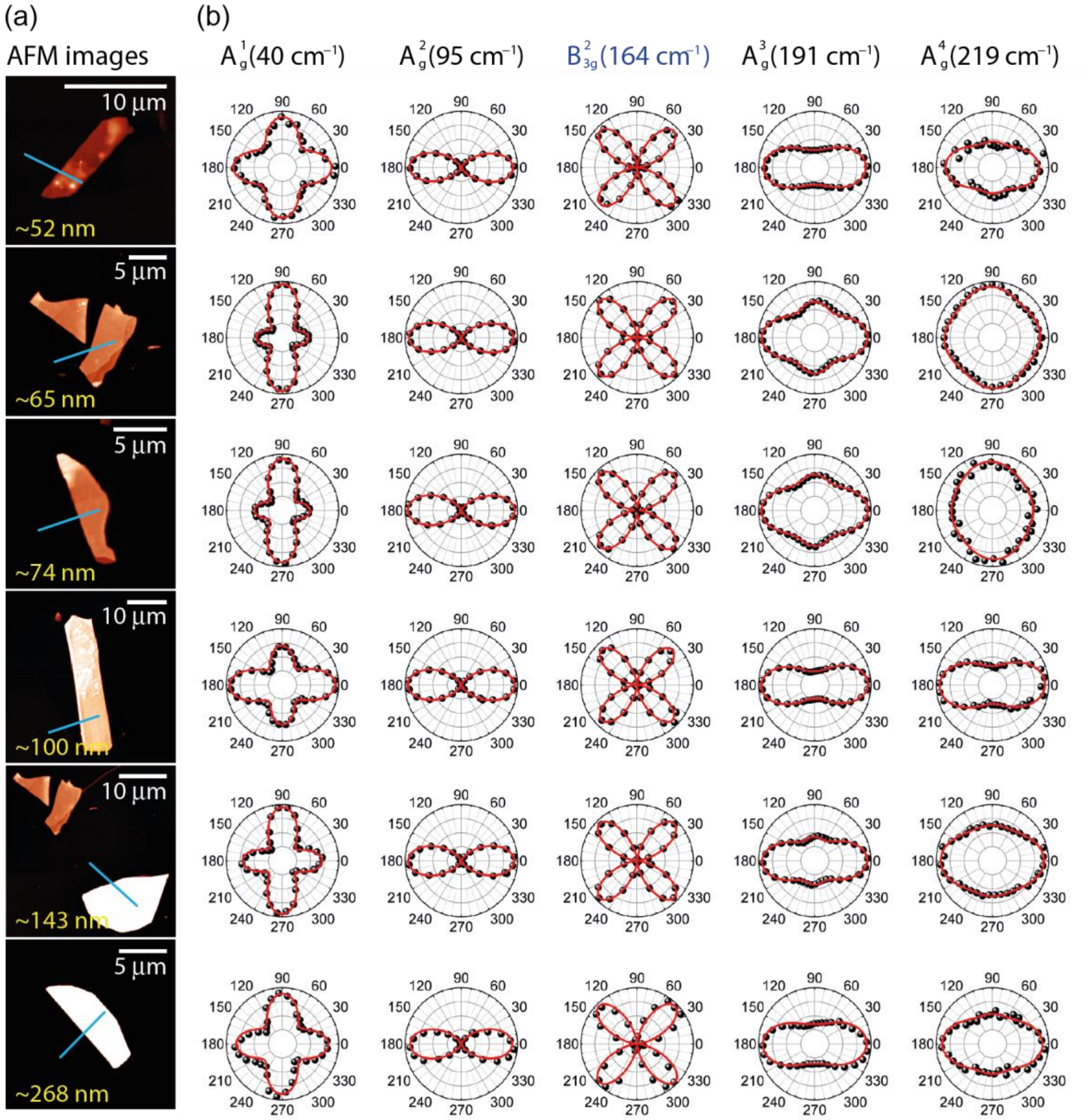

Figure S14. Thickness dependence of Raman modes of several exfoliated SnS samples on SiO_2/Si (280-nm) substrates measured by using 632.8-nm excitation wavelength in parallel polarization configuration. The polar angles are adjusted so that the armchair direction corresponds to 0°. The blue line in each AFM image indicates where the AFM profile was taken to measure the sample thickness.

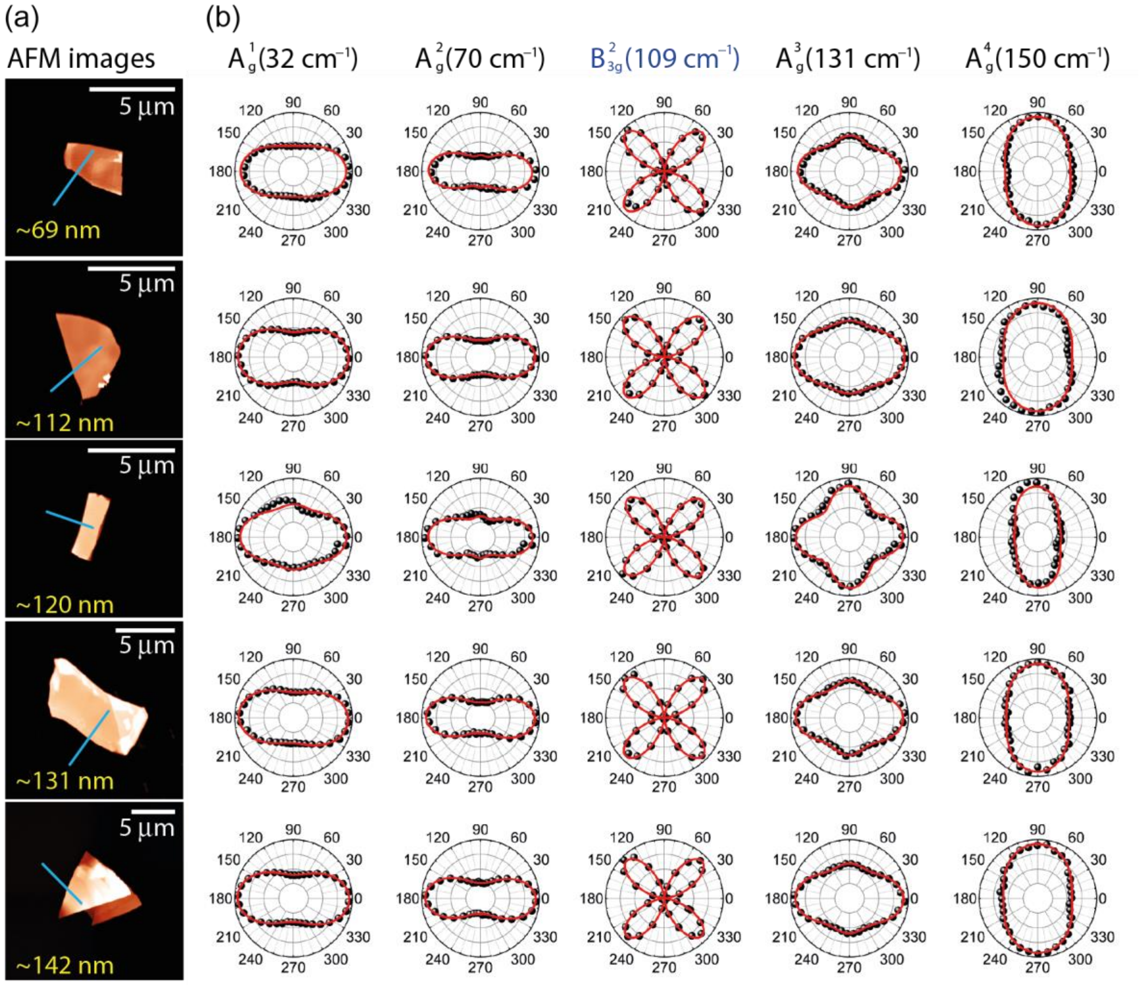

Figure S15. Thickness dependence of Raman modes of several exfoliated SnSe samples on SiO_2/Si (280-nm) substrates measured by using 632.8-nm excitation wavelength in parallel polarization configuration. The polar angles are adjusted so that the armchair direction corresponds to 0°. The blue line in each AFM image indicates where the AFM profile was taken to measure the sample thickness.

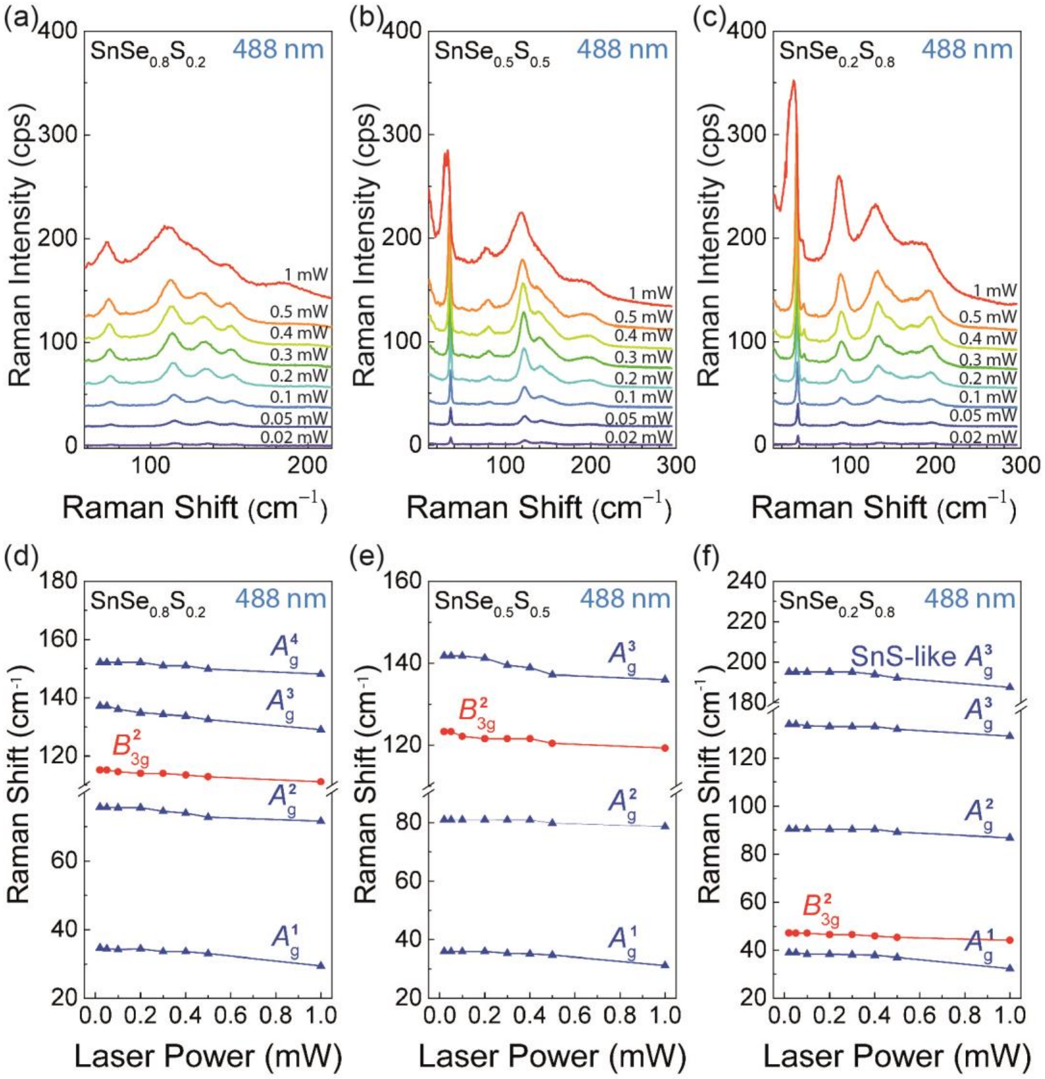

Figure S16. (a-c) Laser power dependence of the Raman spectra of the $SnSe_{(1-x)}S_x$ (x=0.2, 0.5, 0.8) single crystals measured in parallel polarization with the 488-nm excitation wavelength. (d-f) Raman shift of several modes as a function of the incident laser power.
